# Strengthening national capacities for researching on Social Determinants of Health (SDH) towards informing and addressing health inequities in Tanzania

**DOI:** 10.1186/s12939-016-0308-x

**Published:** 2016-02-09

**Authors:** Sally Mtenga, Irene M. Masanja, Masuma Mamdani

**Affiliations:** Ifakara Health Institute, Po Box 78373, Dar es Salaam, Tanzania

**Keywords:** Social determinants of health, Public policies, Health inequities, Research capacities, Research systems, Perceptions, Knowledge, Qualitative

## Abstract

**Background:**

Tanzania’s socio-economic development is challenged by sharp inequities between and within urban and rural areas, and among different socio-economic groups. This paper discusses the importance of strengthening SDH research, knowledge, relevant capacities and responsive systems towards addressing health inequities in Tanzania.

**Methods:**

Based on a conceptual framework for building SDH research capacity, a mapping of existing research systems was undertaken between February and June 2012. It involved a review of national policies, strategies and published SDH-related research outputs from 2005 onwards, and 34 in-depth interviews with a range of stakeholders in Tanzania.

**Results:**

The conceptualization of SDH varies considerably among stakeholders and their professional background, but with some consensus that it is linked to “inequities” being a consequence of poverty, poor planning, limited attention to basic humanity and citizenship rights, weak governance structures and inefficient use of available resources. Commonly perceived SDH factors include age, income, education, beliefs, cultural norms, gender, occupation, nutritional status, access to health care, access to safe water and sanitation and child bearing practices. SDH research is in its infancy but gaining momentum. In the absence of a specific “SDH portfolio”, SDH research is scattered and hidden within disease specific, poverty-related research and research on universal health coverage. Research is mainly externally funded, which has implications on the focus of context specific SDH research, national priorities and transfer to policy. This create mismatch with population and research capacity needs.

**Conclusion:**

Most research analysis in the country fails to consider the context specific structural determinants of health and inequities towards a broader understanding of existing vulnerabilities. The challenge is on promoting a culture of critical inter-disciplinary research and analysis that is central to SDH research. Establishing a system to promote collaboration across sectors and strengthen collective capacities for individuals and institutions researching in SDH will augment existing SDH research initiatives and better inform appropriate intersectoral policies towards addressing prevailing health inequities across the country.

## Background

Health inequalities persist both between and within countries [1]. In most cases, these inequalities are a result of health problems attributable to the different social conditions in which people live and work, such as lower incomes, poor education, poor housing, employment conditions; often referred to as the social determinants of health [SDH]. Irwin et al. [2], all of which are avoidable, unnecessary, and unjust [3–5]. In this paper the social determinants of health concept is based on the WHO-CSDH framework which defines the social determinants of health as: “the conditions in which people are born, grow, live, work and age and seem to encompass the social, economic, political, cultural and environmental determinants of health” [4].

Health inequities are the systematic differences in the health status or in distribution of health resources between different population groups. Health inequities are also avoidable, remediable, unfair and unjust and can be addressed by using resources in a different way [6]. Maternal mortality for example, is a key indicator of health inequity, with wide gaps between rich and poor, both between and within countries [7].

Research evidence compiled by the report of the Commission on Social Determinants of Health [8] showed that improving the health of populations, in a sustainable way, depends not only on improving access to and quality of health services, but also on understanding the underlying causes for inequities in income, power, opportunities, educational level and other social and political factors shaping vulnerability to infectious and non-communicable diseases, lifestyle choices and health behaviour [8].

The consequences of health inequities are particularly dramatic in low- and middle-income countries [9]. Besides economic inequalities [10], social and geographic factors such as gender, race, rural/urban residency and ethnic background also contribute to the large differences in health status and the exclusion of some groups from health services [11]. Inequities also persist between women and men, in part because they are different biologically, in terms of their sex and reproductive health needs [12]; but also because of differences in access to and control over resources in decision-making powers, as well as the roles and responsibilities that society assigns to them [13].

Gender inequalities are an important dimension of wider inequalities in health and health care. Gender intersects with several other inequalities economically, racially, culturally and a number of other social markers, which together contribute to the large differences in health status and the exclusion of some groups from health services [14]. The 2008 World Health Report placed health equity as the central value for the renewal of primary health care and called for priority public health programmes to align with the associated principles and approaches [15]. Besides being accepted as a human right, health represents an important factor for economic and social development [16]. The increasing inequities between and within countries come at a significant financial cost to societies. Persistent inequities slow development [17]. Marmot [17] pointed that globally, there have been some impressive improvements in health, but we still have a long way to go to correct both between and within country health inequalities [18].

### Inequalities and inequities in Tanzania

Tanzania’s social and economic development is challenged by sharp inequalities – between and within urban centers and rural areas, and also among different socio-economic groups. Economic disparity is compounded by unequal access to essential basic services and employment opportunities, and challenged by the gender imbalance of labour and structural and social norms, as well as unequal power relations. While, Tanzania has made significant progress towards achieving global and national targets in key areas of well-being, particularly in health and education, these achievements risk being undermined by persistent poverty. Besides economic disparities, poverty has been linked to health vulnerability. The results of a randomised control trial conducted in Tanzania found a potential benefit of economic incentive on HIV risk behaviour change among young adults living in rural area [51]. Over the years the demographic and Health Survey [52], and the Malaria and HIV indicator surveys (THMIS, 2012) reports in Tanzania have been presenting the contribution of various SDHs i.e. education, income, residence, age, geographical location, gender on differential health outcome including reproductive health, malaria and HIV/AIDS.

There have been widening wealth and geographical inequalities in child survival, and more limited progress in social and regional differentials on areas that are key to improving child and mother’s survival [19]. Sustained economic growth (of around seven percent over the last few years) has not benefitted the majority of the population. Rural inhabitants, poorer, less educated and women continue to be disadvantaged [20–28]. The broader contribution of social ingredients to health is neither well understood nor established in a contextualized environment. Health equity concerns often remain limited to improving physical (geographical) access to health care.

Research is essential to tackle health inequities and take social determinants of health into account for concrete actions. However, despite available evidence on the link between SDHs and health inequities, health equity and SDH concerns are not yet mainstreamed across all sectors, departments and tiers of governments and in the broader development agenda.

Exchanging experiences and linking expertise across institutions, sectors and countries in order to better understand what works and what does not within each context is essential for strengthening research capacities, health systems and contextually relevant approaches to pressing health challenges facing individuals, families, communities and nations at large.

Given the critical importance of SDH research in informing appropriate policies for addressing health equity and health inequality, we explored the SDH landscape in Tanzania. Specifically the conceptualisation, nature, extent and reach of SDH research, supporting national systems and processes for SDH research.

## Methods

Based on a conceptual framework for building SDH research capacity that was developed by the SDH Network [29], we designed standard guidelines for mapping research for SDH and health inequity to understand the SDH landscape in Tanzania. This included the conceptualisation, nature, extent and reach of SDH research, supporting national systems and processes for SDH research [30].

Mapping was carried out over a 5 month period from February to June 2012. It was informed by two sources of data: a review of available information on published SDH-related research outputs from 2005 onwards, research systems (policies, strategies, programmes) and in-depth interviews with 34 key informants from a cross section of institutions; ten national research, policy or advocacy institutions; one national coordinating institution; six government ministries and centres; and four development organisations (see Appendix 1, Table 1).

In the absence of a specific SDH research portfolio, we decided to review literature and existing strategies, policies and plans related to key health priorities as defined by the National Institute of Medical Research and that is based on the national burden of disease, policy commitment, relevance and urgency. This gave us an overview of the type and range of SDH-related research that has been carried out in the country since 2005, including underlying reasons behind existing health inequities and affected population. As well it helped to identify the sectors and actors (government, research institutions or research groups and non-governmental organisations (NGOs) involved in undertaking, communicating and advocating for the implications of the specific research findings.

The review guided the selection of participants for key informant interviews that were purposively and conveniently recruited [31], and limited to Dar es Salaam based institutions due to cost and time constraints. The mapping process used guiding questions (Appendix 3, Table 2) to better understand how SDH is understood and conceptualised amongst the key informant interviews, the extent to which SDH research addresses national research needs, as well as systems in place to support the process (see Table 2). Two pilot interviews were performed in order to test the guide.

The interviews were conducted by two qualified qualitative researchers in the privacy of stakeholders’ offices. One of the interviewing researchers was responsible for compiling detailed notes after each interview (including quotes to illustrate interviewees’ voices) and summarising the content of the interview. Data was manually coded using Microsoft word/ excel, applying thematic content analysis, identifying recurrent themes that formed a cluster of linked categories containing similar meanings.

To validate findings, we triangulated data across respondent groups and looked for supporting documentary evidence, where available. The fact that we were seeking perspectives of individuals from different organisations also enhanced confidence that we are presenting a balanced account. The draft Tanzania mapping report was shared and discussed with country stakeholders during a half-day workshop and their feedback was incorporated in the final version.

Ethical approval was received from the Ifakara Health Institute institutional review board approval number: 1BI1IRB/38. Verbal informed consent was obtained from all respondents. The information was anonymised and confidential.

## Results

Here we present an overview of the SDH landscape in Tanzania based on the results of the mapping exercise, with a focus on the following key themes: the conceptualisation of SDH and its role in addressing health inequities; SDH research activity, priorities and capacity needs; national systems in place to support and promote SDH research; and the outreach of SDH research [32].

### Conceptualisation of SDH and its role in addressing health inequities

Based on stakeholders opinions, when discussing SDH and health inequities in the Tanzanian context, one must at least consider the following: socio-demographic factors, harmful practices and beliefs, poor living conditions, psychological, access to public services, health seeking behaviour, access to information, coping strategies in the face of a shock or chronic poverty, migration, rapid urbanization, changing lifestyles, social and cultural norms, unequal power relations and food security (Appendix 4, Box A).

‘SDH’ is also considered as relatively a new concept and its understanding varies considerably depending on ones discipline and professional background, but with an overall consensus that it is linked to existing “inequities and inequalities”.

Research from Tanzania also suggests that poverty is the key social determinants that affect health status and health outcomes [33–36]. In Tamasha’s study of specific educational needs of HIV (Human Immunodeficiency Virus) positive learners for example, poverty is cited as a limiting condition at almost every level of the HIV positive child or youth learners’ experience and is a key influence in the attitude and actions of many parents. Poverty is linked to school dropout and hunger, the latter posing a real problem for children on ARV (antiretroviral) treatment [37]. As noted by one young orphan “*We are tempted to enter love affairs to get money to pay for our school requirements. When a girl is propositioned and life is tough, she cannot refuse. Those who want to have sex with us are not boys or our own age, but adults*.”

SDH matters much more for poorer people. Compared to the better off, the poor are more likely to lack access to quality public and social services, have inadequate diet, suffer poorer health and need more health care, but often get less. Many poor areas and individuals have limited social capital, and limited access to social networks essential to obtain resources and overcome periodic domestic crises.

### SDH research system activity, priorities and capacity needs

SDH research activities are scattered and embedded within ongoing disease-specific and social-development related research. The recurring SDH researched areas are inequality, reproductive and sexual health (including HIV/AIDS), malaria, gender issues, socio-economic factors, poverty, and the health system itself. Future areas of concern include environmental health, urbanization and non-communicable diseases linked to changing lifestyles and dietary habits.

Many research disciplines are engaged in SDH research, including epidemiologists, social scientists, medical anthropologists, statisticians, economists, political scientists, demographers, public health specialists, geographers, public policy analysts, urban-regional planners, gender activists, advocacy and communication specialists. Quantitative research dominates, though there is a gradual appreciation for qualitative research. Interdisciplinary research towards a critical multidimensional “SDH” view to existing inequities is beginning to gain momentum.

Exploration of the social determinants of health raises a number of methodological issues, chiefly related to measurement, which is not a problem in the more quantitatively oriented disciplines such as epidemiology. The methods, definitions and scale of operation used in SDH research are distinct from those used in epidemiology. Community-based research for example, is essential in SDH research to contextualize research and provide insights into determinants of diseases at the local level, in the setting where interventions actually have an impact on health. For example, what do people do when infected with malaria and why? What are their health care seeking behaviour patterns? These processes are difficult to measure objectively, but the dynamics of change can be analysed rigorously and available information can be used for designing appropriate health promotion messages.

The SDH agenda in Tanzania is still in its infancy and there is considerable scope for further research on SDH and health inequity. In the last few years, there has been an increase in the quantity and an improvement in the quality of studies on the existing associations between health of populations and inequalities. Increased research capacities would allow for better linkages of these areas, placing them in the wider context of SDH. Appendix 5, Table 3 below summarises some of the specific research capacity needs as identified by the interviewed stakeholders. Appendix 2 provides a description of some SDHs related studies conducted in the last few years.

In balance, there is no shortage of individual research skills. The core issue is one of coordinating and maximizing the use of available research skills, getting them to work together, generating a healthy discourse and instilling some critical ‘out of the box’ thinking towards defining research questions. This linkage is necessary for moving beyond the dominant focus on ‘averages’ for meeting global targets, and engaging in some meaningful nationally relevant contextual specific inequity analysis disaggregated, for example, by geography, gender, age, etc.

### National systems to support and promote SDH research

Tanzania has a longstanding commitment to health equity. The concern for inequalities and inequities is central to Tanzania’s Vision 2025, the country’s long-term development agenda towards achieving high quality of livelihood for all Tanzanians by 2025 [38]. The first Five Year Development Plan (1964–1969) aimed to ensure that every Tanzanian lives within 5 km of a health facility, which was a bold move towards equity [39]. The National Strategy for Growth and Reduction of Poverty (2011–2015), the national framework to achieve Vision 2025 recognizes the multidimensional nature of poverty, and adopts an outcome based approach which requires all sectors to contribute to the poverty reduction agenda [38]. The Arusha Declaration of 1967 was the birth of the primary health care concept even before the 1978 Alma Ata Conference on Primary Health Care [40].

The National Health Policy of 1990, revised in 2003, health sector reform proposals and successive national health sector strategic plans have given major focus to reducing inequalities in health [41]. The most recent strategy focuses on the health sector ‘getting its own house in order’ for equity. SDH already has an important place in the forthcoming national health sector strategic plan [42]. The draft Social Health Protection Framework for Tanzania has defined health related sectors (e.g. employment, education, housing, food, agriculture, infrastructure, etc.) in which SDH strategies are adoptable and it clearly spells out the policy directions touching on SDH. Less clear are the legal and constitutional provisions that will support strategies on health, or entitlements on health and its determinants or health care. Despite its critical importance, the Constitution of the United Republic of Tanzania of 1977 does not include the right to health or health equity in its bill of rights [43]. Despite the challenges, several good policies exist, but, the problem is one on implementing them; i.e. putting policies into practice, effectively and equitably [44].

A number of research and advocacy organizations in Tanzania are working on ‘SDH related’ issues pertaining to health and its inequities, some with a focus on research, others working actively towards dissemination and advocating for change, and a few on program implementations. The core national institutions for health research are the Ifakara Health Institute and the National Institute of Medical Research in Tanzania. The Tanzania Commission for Science and Technology (COSTECH) that is under the Ministry of Communication, Science and Technology, is the principal advisory organ to the Tanzanian Government and is responsible for coordinating all research and development in the country. In practice, coordination of research is reported to be difficult and unrealistic possibly because of weak infrastructure and an externally driven research agenda, and also not desirable.

“*Coordination of all research carried out within Tanzania is not, in my opinion, possible nor desirable – let 100 flowers bloom. At the same time, it would be useful to strengthen mechanisms of sharing information and documenting all the different kinds of research which are carried out*.” Policy Analyst.

National investment in SDH research is marginal. Strengthening and sustaining national research capacity in SDH requires a national commitment to building research systems that will allow for researchers to respond to national priorities.

“*..budget allocation to R&D as a proportion of GDP Tanzania is expected to reach 1% by 2015..South Korea’s R&D expenditure as a proportion of GDP is 3.5…. implies that if the allocation of research is bigger, the government really expects researchers to feed them with well evidence based research to policy process and practice….”* Researcher, from TAKNET, 01/11/2011.”

There is however, some concern regarding the absorption capacity of existing research institutions for carrying out good quality research.

### SDH research outreach: informing policy and practice

A senior policy analyst describing challenges in power relations noted that “*there is also a lot of confusion as regards the term “policy makers”. Often researchers and other actors in the development arena define and recognize on National policy makers [*at central level within the Ministries] *as the policy makers, totally oblivious to the reality that there are local policy makers at district level who in fact wield the power to make or break, regardless of what national policy dictates!”*

*“[….] what use is it for a conference or a reputable journal published in New York to address the rise in malnutrition rates in a district in Tanzania when the local leaders and local policy makers there are unaware of the situation? Who will address the challenge in the district - the conference, the journal, or some unseen force? Unless and until researchers learn to provide timely feedback at local level, learn to engage with the target audiences in planning for and implementing “doable” interventions to address the findings, however small, conferences will continue to be held, papers will continue to be published in reputable journals, but to zero results for the poor Tanzanians and others in the same category across the globe [….]”*

Some policy makers report that the link between researchers and policy makers remains a weak one and research is of limited value in influencing policies at all levels. There is reportedly a “*huge disconnect between researchers and implementers*”, in particular “*at local level where most of the data is generated. Researchers’ often lack skills and/or time in disseminating research outside academic circles. They fail to appropriately “package” research findings that consider the needs of different policy audiences*”.

Choosing appropriate techniques for sharing and communicating research is essential. Getting it out to the policy makers and into the media in innovative ways, seizing windows of opportunity and capitalizing on networks and personal links is critical. As noted by one activist:

*“[..] when it comes to turning research into policy and practice, I am a big believer in getting out of the academics’ comfort zone. That means less research reports and policy briefs, and using a more public approach, heavy on dissemination through the media (including social media) and trying to build a small network of influential political allies.– more through the informal political scene than through formal channels of dialogue and/or research […]”*

The recently adopted national research and development policy and strategy (2010) is expected to improve the use of research evidence in the policy process and practices [45]. However, even well-developed research findings may not be acted upon if the political climate is not conducive to change. A policy analyst commented;

*“..we already know the underlying reasons behind health inequities… can research make a difference?… addressing ‘inequities’ is challenging the ‘status quo’, the power base… we can only shift the scene when planners, policymakers, civil society, etc. actively recognize that social determinants shape equity/ barriers to services and opportunities, and that these are the drivers of poor health and the absence of well-being. It is to a large extent an issue of political will, and the translation of political will into formal expectations of planners and service providers”*

Context plays a crucial role in shaping how research evidence is used. Available evidence from some ‘best practice’ examples suggests that a number of factors are critical in shaping the extent to which research is used; that is, credible evidence, an influential leader/ champion, informed citizens and public debate, mobilization of resources, strategic alliances, coalitions and framing research evidence in ways that are attractive to policy makers are effective modes of influencing political priorities.

According to one policy maker, “*the main constraints in addressing SDH and health inequity are several but the main ones include negative forces that come with the heavy reliance on external funds, lack of engagement and participation of key stakeholders in decision making due to their ignorance as regards their rights and obligations and conflicting ideologies between politicians and planners (and pending on which technocrats are involved, politics usually overrides planning)*.”

A number of steps towards addressing existing inequities have been identified, including mobilization of “own” resources to address national priorities; strong governance structures for better coordination and collaboration between health and other “SDH” stakeholders, effective use of available resources; as well as expanding citizen awareness, synthesizing of available research findings within the SDHs framework, engagement with policies and budget process towards ensuring increased basic human and citizenship rights and obligations and allocation of resources according to need.

## Discussion

Strengthening research capacities and building human capabilities has been high on the national agenda. There is nearly unanimous consensus among all stakeholders about the vital role of research capacity in bridging the “know-do gap” and in addressing inequities in health research. But as in several other developing nations, there however continues to be much discussion about “*what kind of capacity* is important for research, *whose capacity* should be strengthened/built and *how best* to strengthen it” [46].

The Commission on Health Research for Development in 1990 commended the need to build sustainable research capacity: *“Building and sustaining research capacity within developing countries is an essential and effective means of accelerating research contributions to health and development. Nurturing individual scientific competence and leadership, strengthening institutions, establishing strong linkages between research and action agencies, and reinforcing national institutions through international networks are all important elements of capacity building”* [47].

Taking into account that health determinants vary between and within countries and regions [8], it is necessary to contextualise internationally available evidence, conduct local analysis and strengthen the evidence base on health inequity, the social determinants of health, and what works or does not, in specific circumstances [48]. A variety of analytical frameworks to describe SDH and guide public health and other policy measures have been developed, such as one established by WHO [9] focusing on the analysis, intervention and measurement on five levels (socio-economic context and position, differential exposure to risk factors, differential vulnerability, differential health care outcomes and differential consequences).

While the important determinants of health are similar across different regions of the world, the differences and local specificities, points to the need to develop locally specific approaches and to contextualise methodology, tools and evidence. Tanzania needs to prioritise strengthening of individual and institutional capacities for carrying out SDH-relevant research. National support for core institutional funding and capacity strengthening is fundamental to sustaining a broader SDH research and policy portfolio that addresses national priorities and requires long and systematic efforts [49]. To make maximum use of available resources and not weaken existing research systems, research capacity building initiatives should build on existing ongoing initiatives and avoid parallel structures. A repository (for example, an ‘SDH clearing house”) of published and unpublished SDH research that is easily accessible by all interested stakeholders will reduce duplication of studies and allow for better use of existing knowledge.

It will also facilitate a synthesis of available SDH findings to date and inform the future research agenda: what we know and what more do we need to know. Additionally, it will also foment collaboration and partnerships within and among institutions and potentially promote a critical mass of interdisciplinary researchers addressing SDH related inequities.

A better understanding of the SDH concept amongst all stakeholders is a pre-requisite to effective implementation of SDH research and the extent to which the key SDHs are explored and addressed in various contexts. In order to reach the widest audience and especially policy-makers at all levels, research needs to be ‘demand driven’ and results need to be appropriately ‘packaged and marketed through diverse channels to ensure effective uptake of findings. Findings from such research can be used to initiate a public debate around national inequities, to inform and empower communities to demand basic human rights, and to provide impetus for appropriate policies and programs.

Researchers often do not see or recognize the social, political and economic context and the many forces at work in the policy environment that provide challenges to integrating evidence into policy and practice. The “*how and when evidence is used often depends upon the political agenda and ideology of the government of the day, not on the nature of the evidence, however compelling*” [50]. The key is to forge strong communication networks, promoting collaborative interdisciplinary research partnerships, between public and private institutions, and within and across research institutions, as well as with funding agencies, advocacy groups and policy makers at all levels and getting it onto the national political agenda.

Tanzania’s developing and rapidly growing economy is under increasing public pressure to ensure that economic growth leads to social opportunities and improved access to education, health and other public services. Tanzania has many opportunities for improving health equity, including its political stability, socially cohesion, a decade of consistent economic growth, low income inequality, improved wage to GDP ratio and recent improvements in job creation. It graduated from the low to middle human development group of countries, primarily due to investments in education and health, with many decades of policy support for health equity, including the adoption of the PHC approach a decade before Alma Ata [19].

In sum, the role of public policy is crucial in determining national welfare [50]. There is increasing recognition that strengthening and promoting capacities for researching the SDH is necessary to design context specific strategies and policies to tackle current complex health problems and address inequities. The involvement of multiple sectors is a prerequisite to reduce health inequalities since health, social, financial, economic, and transportation policies are all needed to improve the social determinants of health.

## Conclusion

Tanzania has made great strides in recognizing the importance of SDH, as noted by its plans and policies, and a growing body of current research on SDH in the country. Over the last few years, policy makers, development partners, practitioners, activists, and academics working in applied research are recognizing the importance of contextualizing research to better understand what works, how, where, why and why not. There is a clear recognition that in the process of scaling up, even the most successful pilots need to be adapted to the specific context and the needs of the communities involved.

Research is not only valuable in itself but as a driver of wider social change. Research based on co‐production of knowledge with different sets of stakeholders has the potential of contributing to better policy and public debate. Initiatives that resonate with and respond to broad public concern are more likely to gain traction, exercise accountability and be sustainable.

Priority should be given to establishing systems to capture nationally relevant research activities that inform context specific SDH and develop individual and institutional research capabilities for action on the SDH and health inequities.

### Limitations

This mapping report is not comprehensive or exhaustive. It does not reflect all SDH related activities in the country. It is based on a desk review of some national policies, strategies and programmes and published SDH-related research outputs from 2005 onwards, complemented by in-depth interviews of a select few individuals from a cross section of Dar es Salaam based institutions.

**Table 1:** List of stakeholders interviewed during the mapping activity

National level coordinating system	Government ministries or centres	National Research/Policy/Advocacy Institutions	Development partners
COSTECH (1 person)	MDFN (1 person)	AMREF (1 person)	SDH ( 2 persons)
	MCDGC (2 persons)	DARAJA (1 person)	Irish Aid ( 2 persons)
	MoHSW (1 person)	IHI (7 persons)	UNICEF (1 person)
	MoFEA (2 persons)	MUHAS (2 persons)	WHO (1 person)
	TACAIDS (1 person)	NIMR (2 persons)	
	TFNC (1 person)	REPOA (2 persons)	
		SIKIKA(1 person)	
		TAMASHA (1 person)	
		TGNP (1 person)	
		TWAWEZA (1 person)

**Table 2:** General Trends in published and ongoing research related to SDH and Health Inequity since 2005 - some examples reflecting the diversity

***Malaria***	Bruno P MMbando et al. 2011. Spatial variation and socio-economic determinants of Plasmodium falciparum infection in north eastern Tanzania. Malaria Journal, 10:145
	Emmanuel et al. 2007.
	Kahigwa E. undated. Social-cultural factors that influence the implementation of malaria prevention diagnosis and treatment interventions in Tanzania. www.ihi.or.tz
	Manuel W, Hetzel et al. 2008. Malaria risk and access to prevention and treatment in the paddies of the Kilombero valley, Tanzania. Malaria Journal 2008.
	Masanja H. Information and beliefs about malaria and bed net usage in Rufiji DSS: www.ihi.or.tz
	Rashid A Khatib et al., Market, Voucher, subsidies and free nets combine to achieve high bed net coverage in rural Tanzania. (IHI)
	Smithson P. 2009. Down but not out: the impact of malaria control in Tanzania. Spotlight, 2. Dar es Salaam: IHI.
***HIV/AIDS***	Fox A. 2010. Social determinants of sero-status in Sub-Saharan African. An inverse Relationship between Poverty and HIV. Public Health Reports, Supplement 4,Volumbe 125.
	Kessy F, Mayumana I and Msongwe Y. 2010. Widowhood and vulnerability to HIV and AIDS-related shocks: exploring resilience avenues. REPOA Research Report 10/5.
	Mtenga S and Nathan R. (2005) Reaching the poor with Voluntary Counseling and Testing for HIV/AIDS and Treatment of Opportunistic infections. Presentation at the INDEPTH-Annual General Meeting in Burkinafaso

**Table 3:** Guiding questions

• How is SDH understood within the Tanzanian context? What are the key SDH and causes of inequities in health? • What are recent and planned SDH research activities, networks (collaborations), capacity priorities and needs at national level? • What is the capacity to coordinate, produce, use and generate demand for research on SDH and health inequity? • Who are the key players shaping the SDH research and policy agenda? • What are the national SDH and health inequity research priorities to guide researchers and trainers at institutional level? • Which kinds of collaboration or exchange programmes exist and how do they influence research and research policy? • How do power relations interfere with the development/prioritization of the health and SDH research agenda? • To what extent is health and SDH research determined by the financing mechanisms? • To what extent is SDH research constrained by the lack of adequate research infrastructure and coordination – HR and research skills and disciplines, resources, research management capacity, locally relevant research capacity building tools and initiatives, etc.? • To what extent are decision-making processes institutionalized? • What mechanisms are in place to share and disseminate research findings, ensuring that they feed into policy-making bodies?

**Table 4:** Stakeholder’s conceptualisation of SDH and health inequity

*“For rich countries at least, the landmark Black Report in the UK clearly stands out; it argues that inequality has a strong and independent effect on the health of individuals and entire nations. I don’t know how this would apply in a poor and not-so-unequal country like Tanzania. Here I can think more in terms of things like these: mothers in Pemba who allegedly refuse being immunized against tetanus because they think it will make them infertile..; community members in Lupalilo, Makete, who said it was fine, and normal, for a woman to plough the field until the very final stages of their pregnancy because that’s their job and anyway it was their responsibility if they got pregnant in the first place; family and larger societal pressures that force a girl to marry young rather than complete school, despite the known risks to her health from early pregnancy; economic forces and social pressures that force new mothers to go back to the field just weeks after delivery, thereby compromising their ability to care for their health and breast feed their newborns/infants; traditions that impose FGM (female genital mutilation) upon unsuspecting girls and adolescents, endangering their health; beliefs, such as witchcraft, that put the life of albinos at risk; and if we count economic forces as being part of SDH, then another example would be the poor fishermen who engage in dynamite fishing, are maimed and end up as beggars in a Dar es Salaam street corner.”* *“SDHs are the direct and non direct factors related to health outcome, the enabling and pre-disposing factor, individual and structural/ system factors; moving beyond the health sector to all social aspects that may in one way or another contribute to one’s well being, including issues related to empowerment, housing conditions, lifestyles, economic and social status, having clean water, food, education, as well as infrastructures for transporting food, accessing health care. Factors that are associated with either negative or positive outcome of health.”* *“In line with our transformative feminist perspective on health, I consider SDH to be rooted in the dominant structures of power in society, including economic, political, cultural and ideological…. understanding that health includes not only the absence of illness, but also a sense of well-being, security, dignity, happiness and self-fulfillment, different groups of people have different levels of access to the basic resources necessary for health, well being, and sustainable livelihoods. These include access to dignified and secure employment and livelihoods with a livable income, and the means to acquire employment and livelihoods – be they land, water, markets, information, credit; and the prerequisites for employment such as formal education and employment such as a formal education and employment experience.”* *“Girls and women also experience, on a daily basis, a variety and a barrage of emotional and sexual abuse, discrimination and oppression which eats away at their self-esteem and deprives them of security and safety, as well as physical health……on the other hand, however, SDH also include the way in which marginalized girls/ women, and their communities, respond to the existing structures of power at all levels …women have organized themselves in HISA groups which not only provide a safe space for savings and loans, but also provide mutual support mechanisms in times of illness and death. Community groups along with national/ local NGOs increasingly participate in performance tracking to demand accountability from health delivering institutions…however, there is not yet a high level of community consciousness about basic human and citizenship rights, let alone health rights, nor the development yet of a strong popular movement for health as a basic human right…”*

**Table 5:** Research capacity needs

• Enhancing qualitative social science research skills • Strengthening analytical skills at all levels, enabling researcher analysts and community animators to link micro and macro issues • Promoting public discourse about the SDH concept • Synthesizing available research findings from an “SDH and inequity” perspective that would guide their next “research/ policy/ communications” agenda • mainstreaming ethics in research • Determining the interrelations between general aspects such as social, economic and political factors and the way through which these factors affect the health of groups and individuals (will for a better understanding of correlations/ dynamics between general wealth indexes of a given society, such as the GDP, and the health indexes) • Using available secondary data to understand the determinants better and designing potential interventions to address inequities.	

## Abbreviations

AMREF: African Medical and Research Foundation, Tanzania
COSTECH: Commission for Science and Technology
IHI: Ifakara Health Institute
MADFN: Ministry of Agriculture, Department of Food and Nutrition
MCDGC: Ministries of Community Development, Gender and Children
MoFEA: Ministry of Finance and Economic Affairs (MoFEA), Department of Planning and Budgeting
MoHSW: Ministry of Health and Social Welfare (MoHSW), Department of Non Communicable Diseases (Mental Health)
MUHAS: Muhimbili University of Health and Allied Sciences (MUHAS), Department of Behavioural Sciences
NIMR: National Medical Research Institute (NIMR), Department of Research Ethics
REPOA: Research on Poverty Alleviation
SDC: Swiss Development Corporation
TACAIDS: Tanzania Commission for AIDS
TFNC: Tanzania Food and Nutrition Centre
TGNP: Tanzania Gender Network Programme
UNICEF: United Nations Children’s Fund
WHO: World Health Organisation
